# Central Nervous System Effects of Early HIV Infection and Consequences of Antiretroviral Therapy Initiation during Acute HIV

**DOI:** 10.3390/v16071082

**Published:** 2024-07-05

**Authors:** Phillip Chan, Serena Spudich

**Affiliations:** 1 Department of Neurology, Yale School of Medicine, New Haven, CT 06510, USA; 2 Center for Brain and Mind Health, Yale School of Medicine, New Haven, CT 06510, USA

**Keywords:** acute HIV infection, primary HIV infection, central nervous system, cerebrospinal fluid, cognitive assessment, neuroimaging, antiretroviral therapy

## Abstract

HIV infection is a multi-organ disease that involves the central nervous system (CNS). While devastating CNS complications such as HIV-associated dementia and CNS opportunistic infection typically manifest years after HIV acquisition, HIV RNA is readily detected in the cerebrospinal fluid in untreated neuroasymptomatic people with HIV, highlighting that HIV neuroinvasion predates overt clinical manifestations. Over the past two decades, increased awareness of HIV infection within the at-risk population, coupled with the accessibility of nucleic acid testing and modern HIV immunoassays, has made the detection of acute and early HIV infection readily achievable. This review aims to summarize research findings on CNS involvement during acute and early HIV infection, as well as the outcomes following the immediate initiation of antiretroviral therapy during this early stage of infection. The knowledge gap in long-term neuroprotection through early ART within the first year of infection will be discussed.

## 1. Introduction 

HIV infection is a multi-organ disease that involves the central nervous system (CNS). While HIV-associated dementia (HAD) [1] and CNS opportunistic infection are typically late manifestations in people with HIV (PWH) with advanced immunodeficiency, HIV RNA is readily detected in the cerebrospinal fluid (CSF) in “neuroasymptomatic” PWH [2], highlighting that HIV neuroinvasion predates neurological manifestations. In the era of highly efficient antiretroviral therapy (ART), PWH who adhere to ART broadly achieve sustained virologic suppression and a considerable degree of immune recovery. To date, HAD and CNS opportunistic infections have become exceedingly uncommon. However, cognitive deficits are present in at least 20% of PWH on suppressive ART, though the exact prevalence is variable based on the criteria used to define cognitive impairment, as well as comorbid and demographic factors [3]. Moreover, despite careful adjustment for HIV control status, cardiovascular disease (CVD) and vascular risk factors, which predict cognitive decline in people without HIV (PWoH) [4,5], remain more prevalent in PWH when compared to PWoH [6,7]. The elevated risk of vascular disease raises concerns about whether PWH on ART would experience accelerated cognitive aging through mechanisms in vascular cognitive impairment (VCI) [8,9].

To date, comprehending the evolution of HIV pathogenesis within the CNS remains a critical undertaking for clinical management and for HIV cure strategies. The former intersects with the expanding aging populations of PWH worldwide [10], where cerebrovascular and neurodegenerative diseases come into play. The latter aligns closely with the growing evidence supporting the CNS as one of the HIV reservoirs [11,12]. HIV DNA is frequently detected in brain autopsies from PWH after years of suppressive ART [13,14,15,16]. CSF viral escape [17], in the setting of detectable HIV RNA in the CSF despite suppression in plasma using commercially available assay, occurs in 5–10% of asymptomatic PWH on suppressive ART [18]. In this regard, knowledge of early HIV pathogenesis is crucial to inform the timing of HIV establishment of persistence and compartmentalization in the CNS.

Nonetheless, the identification of HIV infection at its earliest stages is profoundly challenging. First, symptoms following HIV acquisition are usually transient and can be subtle [19,20]. Second, the diagnosis necessitates the establishment of sophisticated laboratory pipelines that may not be feasible in non-research and resource-limited settings. Above all, characterizing the neurological features of early HIV infection requires a comprehensive approach, combining mood and cognitive assessments, neuroimaging, and preferably, CSF evaluation at the time of diagnosis and longitudinally during follow-up. Over the past two decades, an increased awareness of HIV infection within the population, coupled with the accessibility of nucleic acid testing (NAT) and modern HIV immunoassays, has made the detection of acute and early HIV infection readily achievable.

This review aims to consolidate research findings on CNS during acute and primary HIV infection. Acute HIV infection (AHI) is defined by Fiebig stages I to V (see below) [21], while primary HIV infection (PHI) refers to a diagnosis of HIV infection within the first year following acquisition. The latter will be used interchangeably with early HIV infection in the following sections. The review encompasses post-ART outcomes from cognitive and mood assessments, neuroimaging, and virological and biomarker studies in the CSF, highlighting key differences between PWH who initiated ART during early HIV infection versus those who initiated ART during chronic HIV infection (CHI). Current knowledge gaps regarding ART initiation during early HIV infection will be discussed.

## 2. Search Strategy and Selection Criteria

To identify articles on CNS and early HIV infection, we searched PubMed using the following terms: “acute HIV infection”, “primary HIV infection”, or “early HIV infection”, combined with “central nervous system”, “CNS”, “brain”, “cerebrospinal fluid”, “CSF”, “magnetic resonance imaging”, “MRI”, “neuroimaging”, “cognitive function”, “cognitive assessment”, or “neuropsychiatric.” Only articles published in English were considered. The searches covered the period from 1 January 2005 to 30 April 2024. We supplemented these searches by reviewing citations in identified manuscripts and recent conference abstracts and leveraging our knowledge of the subject.

## 3. When Does Neuroinvasion Begin after HIV Transmission?

After HIV acquisition, a short eclipse phase of 8–10 days ensues, characterized by the general undetectability of HIV RNA in the plasma through commercially available assays [21,22]. This eclipse phase is succeeded by Fiebig stages I to V of AHI, delineated by the sequential identification of plasma HIV RNA, p24 viral protein, HIV-specific IgM, and a positive result on the Western Blot test without the detection of the p31 band (refer to Table 1). The detection of the p31 protein that marks the end of Fiebig stage V AHI ranges from 30–100 days following HIV acquisition [22]. Plasma HIV viremia peaks at Fiebig stage III, with viral load (VL) measurements often surpassing one million copies/mL, compared to levels ranging from 1000 to 10,000 copies/mL during CHI [22].

Owing to the impracticability of tissue biopsy, the detection of HIV RNA in the CSF typically serves as evidence of HIV invasion into the CNS. The RV254 AHI cohort, conducted in Bangkok, Thailand, represents the largest cohort of its kind. Since 2009, the cohort has enrolled over 700 participants, all of whom also commenced ART within days of enrollment and are being followed longitudinally. CSF studies of RV254 participants during pre-ART AHI revealed that CSF HIV RNA was measurable as early as 8 days after the estimated date of HIV exposure [23]. In an RV254 study with 117 CSF samples, the rate of HIV RNA detection was 51% during Fiebig stages I and II. This is compared to 92% during later Fiebig stages III-V, based on an assay with a lower level of detection of 80 copies/mL [24]. In another RV254 study, active viral transcription (spliced viral RNA) was present in CSF CD4+ T cells from all six CSF specimens collected during Fiebig stages III and IV [25]. The finding is consistent with CSF studies of PWH during pre-ART PHI, where HIV RNA can be detected in nearly all CSF samples [26] and continues to be detected in the ensuing years prior to the initiation of ART [27]. These findings indicate that HIV invades the CNS by the late stages of acute infection and persists in this compartment thereafter.

## 4. Neurological Manifestations during Acute and Early HIV Infection

Though HIV RNA in the CSF is detected in entirely asymptomatic participants during AHI, neurological manifestations are often observed during this period, highlighting that HIV neuroinvasion is not clinically silent and suggesting the likely onset of neuropathogenesis. In an observational study that compared neurobehavioral disturbance of 34 individuals before and shortly after HIV acquisition, apathy and executive dysfunction were exacerbated following the infection and were in association with lower CD4+ T-cell counts [28]. Similarly, based on a standardized neurological evaluation, over 53% of 139 RV254 participants in Thailand exhibited neurological signs or symptoms within the first 12 weeks following AHI [29]. The most common manifestations were cognitive complaints (33%), abnormal motor findings (34%) and neuropathy (11%). Moreover, nearly half of these manifestations were recorded during pre-ART AHI at study enrollment. In a PHI cohort of men who have sex with men (MSM) in the US, signs and symptoms of neuropathy were detected at a median estimated infection duration of 3.5 months. This was accompanied by elevations in immune activation, including plasma and CSF measures of neopterin, a biomarker of the cell-mediated immune response [30]. Mood alterations are also common during AHI and early infection. Using Patient Health Questionnaire-9 (PHQ-9) [31] and Hospital Anxiety and Depression Scale (HADS) [32] questionnaires, clinically relevant depression and anxiety symptoms were detected in 55% and 66% of the RV254 participants during AHI [33]. Importantly, the PHQ-9 score, and therefore the depressive symptoms, increased with plasma HIV-1 RNA and neopterin [34], suggesting a biological link between virologic and immune activation processes and mood symptoms. In the aforementioned US PHI cohort, 47.7% of MSM met the Beck Depression Inventory (BDI) criteria for clinical depression at baseline prior to ART and did not have substantial improvement over time, even after the initiation of ART [35].

Neuropsychiatric assessments help characterize cognitive deficits alongside cognitive complaints during early HIV infection. Based on a 4-test battery assessing psychomotor, executive, and motor functions, which included Color Trails 1 and 2, Trails Making A, and non-dominant hand Grooved Pegboard tests, 25–30% of RV254 participants performed one standard deviation below the norm (i.e., z-score < 1) on two or more tests within the battery during pre-ART AHI [36,37]. A few studies have examined the cognitive performance of PWH during the first year of infection. A US-based study compared the cognitive performance of 63 individuals with CHI, 39 with early HIV infection (<52 weeks of infection), and 38 PWoH using a cognitive battery covering seven cognitive domains [38]. While the study was unable to detect neurocognitive impairments in the early infection group statistically, the group exhibited a clear trend toward having an intermediate performance among the three groups, with PWoH performing the best and PWH with CHI the worst. In the Sabes study conducted in Lima, Peru, 87 participants were assessed within 100 days of HIV infection [39]. Using a battery that covered six cognitive domains, participants’ overall cognitive performance, standardized with normative data, was within the normal range before ART. Finally, a study comparing the cognitive performance between 56 Chicago Early HIV Infection cohort participants and 21 PWoH controls revealed worse performance on measures of verbal memory, visual memory, psychomotor speed, motor speed, and executive function in the PWH group [40]. Collectively, these studies suggest that neurological and neuropsychiatric abnormalities can be observed during the earliest phases of infection.

## 5. Intrathecal and Intracerebral Immune Activation and Neuronal Injury during Acute and Early HIV Infection

Accompanying the detection of HIV RNA in the CSF and neuropsychiatric abnormalities, biomarker alterations are readily observed in CSF and neuroimaging studies during acute and early HIV infection. Compared to CSF samples from PWoH, immune activation markers, such as interleukin 6 (IL-6), CXCL10 (IP10), CCL2 (MCP-1), sCD14, sCD163 and neopterin, are overtly elevated in CSF samples collected during pre-ART AHI [41] and PHI [26]. This is accompanied by an expansion of HIV-specific CD8+ T-cells in the CSF, which increases with CSF HIV RNA levels [42]. Moreover, elevated measures of markers of cellular immune activation, including CSF white blood cell count and CSF neopterin, persist and even increase over time in the CNS during early infection prior to the initiation of ART. These findings indicate that immune activation is not a transient local response to initial infection but, in fact, an early and chronic low-grade leptomeningitis and/or encephalitis in the absence of ART [43].

The measurement of axonal injury markers in plasma and CSF, such as neurofilament light chain (NfL) [44], offers an opportunity to interrogate the onset time of neuronal injury after HIV acquisition. A CSF study compared the NfL levels between 32 RV254 participants during pre-ART AHI, 32 PWH during pre-ART CHI and 18 PWoH controls. In the study, only one (3%) AHI participants exhibited an NfL level above the normal cutoff level, compared to 10 (33%) CHI participants [45]. In the study, CSF NfL levels were statistically comparable between AHI participants and PWoH controls, while the CHI group exhibited a higher CSF NfL level than the PWoH controls. In contrast, CSF NfL was found to be elevated in PHI compared to PWoH, with 36 out of 82 (44%) PHI samples demonstrating NfL levels above the upper limit of normal for their age group [46]. Another study compared NfL levels in 143 CSF samples across a spectrum of immunologic and neurologic severities [47]. The study revealed that the frequencies of elevated NfL levels among PWH with HAD, neuroasymptomatic PWH with a CD4+ T-cell count below 50, and those within the first year of infection were 100%, 75% and 40%, respectively.

Subtle volumetric brain changes are also observed during the first year of infection. In a magnetic resonance imaging (MRI) study with 112 AHI and 18 PWoH participants, AHI participants of late Fiebig stages (III–V) exhibited greater volumes in nucleus accumbens and putamen than AHI participants of early Fiebig stages (I–II) and PWoH controls [48]. The changes may represent the acute inflammatory changes in context with the peak plasma and CSF viremia during the late Fiebig stages. In contrast, reductions in total and cortical grey matter (GM), as well as an expansion of the third ventricle, were detected in 43 participants from the Chicago Early HIV Infection cohort when compared to 21 PWoH [49]. Volume losses in the right cerebellum, bilateral thalami, left caudate, and left temporal lobe, and cortical thinning in bilateral frontal and temporal lobes and cingulate cortex were associated with longer duration of untreated HIV infection in another study that included 65 PHI and 19 PWoH participants [50]. Furthermore, lower putamen volume was associated with lower CD4 T-cell count, a lower CD4/CD8 ratio and worse motor/psychomotor performance in cognitive assessments in a volumetric study with 47 untreated PHI participants [51], highlighting the potential linkage between immunological outcomes, cerebral structural change and cognitive function.

The application of magnetic resonance spectroscopy (MRS) [52] and diffusion tensor imaging (DTI) [53] enables the evaluation of brain metabolites and microstructural integrity during early HIV infection. Consistent with the CSF biomarker analyses, MRS during untreated AHI revealed an elevated Choline/Creatine ratio, a sign of cellular inflammation, in the basal ganglia and occipital grey matter when compared to PWoH controls [54]. However, evidence of neuronal injury, indicated by a reduction of N-acetyl aspartate/Creatine ratio, was not observed. Among PWH with PHI, one MRS study that longitudinally followed nine untreated PHI individuals over 6 months reported a sequential elevation of choline levels in the frontal cortex and white matter (WM) [55], whereas another cross-sectional study that included eight PHI and nine PWoH participants reported lower N-acetyl aspartate and combined glutamate/glutamine levels in the PHI group [56].

DTI is a structural measure that evaluates axonal (i.e., white matter) organization in the brain by determining the fractional anisotropy (i.e., directionality) and mean diffusivity of water molecules. In a study that included 49 RV254 AHI participants and 23 PWoH controls, the DTI metrics of the two groups did not statistically differ [57]. Another study compared DTI metrics between 62 PHI and 19 PWoH participants [58]. Although DTI metrics did not differ statistically between the two groups, these metrics were negatively associated with the estimated duration of HIV infection and the levels of blood-brain barrier (BBB) disruption, as determined by the CSF to plasma albumin ratio, in the PHI group. The latter finding indicates the existence of adverse effects of HIV on brain microstructure.

**Figure 1:**
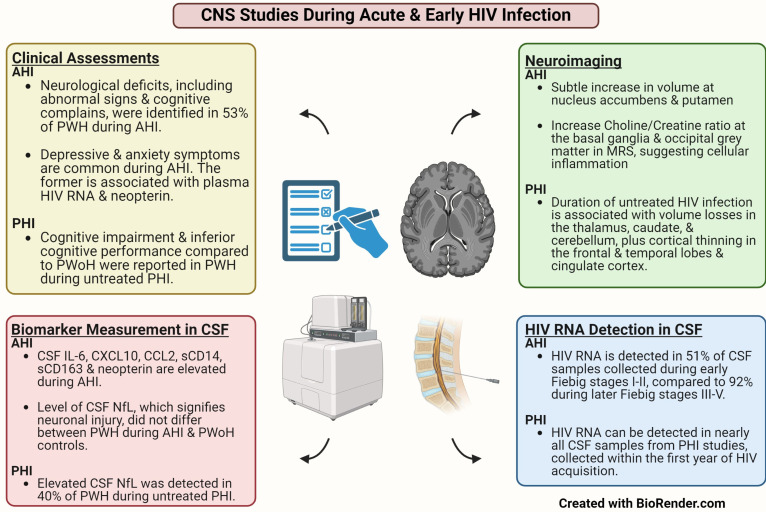
Key findings in Neurological Studies that Focused on Untreated Acute and Primary HIV Infection. Abbreviations: AHI: Acute HIV Infection; CSF: Cerebrospinal Fluid; MRS: Magnetic Resonance Spectroscopy; NfL: Neurofilament Light Chain; PHI: Primary HIV Infection; PWH: People with HIV; PWoH: People without HIV.

Figure 1 summarizes the key CNS findings in HIV cohort studies that focused on the first year of infection. Briefly, CSF biomarker and neuroimaging studies confirmed the parallel onset of intracerebral immune activation along with HIV neuroinvasion in AHI. Additionally, while concrete evidence of neuronal injury and axonal damage is absent during AHI, such damage could be observed in a subset of PWH during the first year of infection.

## 6. Factors That May Alter the Long-Term Neuropathogenesis of HIV

While the duration after HIV acquisition remains a strong predictor of adverse virologic and immunologic measures in acute and early HIV infection studies, there are inter-individual differences. For instance, CSF to plasma VL difference can vary individually during early HIV infection. In general, HIV RNA in plasma is higher than that in the CSF. During CHI, plasma VL is typically 10 times (i.e., 1 log_10_) higher than that in the CSF [2]. In contrast, the VL difference is usually reduced to less than a log_10_ among PWH who have HAD. Further, a considerable proportion of them exhibited CSF discordance, defined by a higher CSF VL compared to plasma [2], indicating an excessive HIV replication within the CNS. In a multi-center study that examined 1018 CSF samples from PWH with PHI, neuroasymptomatic CHI and HAD, the frequencies of CSF discordance were 1%, 11% and 30%, respectively [2]. In AHI, the plasma to CSF VL difference ranges between 2 to 3 log_10_ [24], and CSF discordance has not been reported thus far. However, among those with detectable HIV RNA in CSF, a small subset (~7%) exhibited a reduced VL difference of less than a log_10_ between the two compartments [24]. Notably, HIV RNA in the CSF is anticipated to originate from the systemic circulation due to the absence of local HIV replication by resident CNS cells during AHI. Hence, the reduced VL difference during AHI may imply an escalated transmigration of free virus or infected cells from the systemic circulation into the CSF and CNS compartments.

As many PWH recruited into acute and early HIV cohorts had initiated ART immediately following the initial assessment to achieve optimal long-term outcomes, it remains unclear whether the reduced VL difference between plasma and CFF during early HIV infection leads to worse neurological outcomes, including accelerated HIV compartmentalization in the CNS and earlier development of CNS manifestations in the absence of ART. Cross-sectionally, a reduced plasma to CSF VL difference was associated with a lower blood CD4/CD8 T-cell ratio, higher levels of CSF immune activation markers, and increased odds of concomitant CSF pleocytosis (i.e., high WBC) during AHI [24,59]. While such comparisons fall short of informing the direction of effects, the findings suggest a potential linkage between the degree of HIV transmigration into the CNS, the severity of systemic HIV infection, and an individual’s immunologic response against the infection. Concomitant CNS infection alongside HIV may also alter its transmigration. In a case report, an RV254 participant was diagnosed with asymptomatic neurosyphilis during AHI through CSF examination for research purposes [60]. The individual presented with extreme CSF pleocytosis (WBC > 100), with a reduced plasma to CSF VL difference of 0.81 log_10_. Although the CSF Venereal Disease Research Laboratory test (VDRL) was negative, treatment of neurosyphilis was initiated due to the presence of high blood VDRL titer (1:64), positive CSF T. pallidum hemagglutination assay (TPHA), and extensive meningitis changes in MRI brain scan. The individual underwent CSF sampling and MRI again at 24 weeks post-AHI following neurosyphilis treatment. Despite persistent HIV viremia due to ART non-adherence, MRI abnormalities resolved, and CSF WBC decreased to 10. Additionally, there was an increase in the plasma to CSF viral load difference from 0.81 to 1.60 log_10_, achieving a “normal” value comparable to typical early HIV infection.

Deep sequencing studies evaluate HIV strains in paired plasma and CSF samples to identify unique strains in respective specimens. In the case of acute and early HIV infection, they provide an opportunity to determine the timing of HIV compartmentalization in the CNS. Previous studies confirmed the existence of unique quasispecies in the CSF samples collected within the first year of infection [61,62,63]. Notably, unique quasispecies have been identified in the CSF approximately 3.6 months following HIV acquisition, suggesting HIV compartmentalization in the CSF and possibly the CNS [62]. During AHI, although unique quasispecies were not identified in CSF samples, varying proportions of mostly minor variants could be detected between plasma and CSF, especially among those infected with multiple transmitted/founder viruses [64]. Whether multiple transmitted/founder viruses play a role in the progression of CSF HIV compartmentalization remains to be determined.

## 7. Could ART Initiation during Early HIV Infection Reverse the Systemic and Neurological Changes and Offer Sustainable Neuroprotection?

Among PWH who initiated ART during CHI, years of research confirm the fact that ART initiation during CHI does not completely reverse chronic systemic and intracerebral inflammation. Particularly, PWH on years of suppressive ART persistently exhibited higher levels of plasma immune activation and vascular dysfunction markers compared to PWoH [65,66,67,68]. In the CSF, neopterin remained elevated 2 years after ART initiation at a CD4+ T-cell count above 500 cells/µL [69]. Positron Emission Tomography (PET) of the brain further suggested persistent intracerebral microglial activation in PWH who have been on years of suppressive ART [70,71,72]. Clinically, the landmark Strategic Timing of Anti-Retroviral Treatment (START) trial highlighted a marginal benefit in reducing the risk of cardiovascular complications when ART initiation occurred at a CD4+ T-cell level above 500 cells/μL when compared to delayed initiation until 350 cells/μL in treatment-naïve PWH with CHI [73]. In this regard, longitudinal outcomes from early infection cohorts could inform whether ART initiation within the initial year of infection resolves the residual systemic and intracerebral immune activations that persist in CHI. These findings are crucial for public health policy. If ART initiation within the first year of HIV infection can effectively mitigate residual immune activation and the risk of adverse clinical outcomes, it would justify investing more resources in public education and improving laboratory capabilities for early HIV detection.

Table 2 summarizes the longitudinal outcomes of early HIV cohort studies after ART, organized by testing modalities. In general, cognitive outcomes after ART initiation within the first year of infection appear promising, with a low rate of cognitive impairment. In a group-based trajectory analysis that included 67 RV254 participants who initiated ART during AHI, all identified trajectory subgroups showed improvement in their cognitive test performance over 288 weeks (~5.5 years) of follow-up, achieving composite scores within the normal range on the 4-test battery [37]. Importantly, the subgroup with the worst performance pre-ART showed the greatest extent of improvement during the study period. The finding is different from that of PWH, who began ART during CHI. In the latter case, individuals with CHI who had cognitive impairment pre-ART exhibited an increased risk of cognitive decline compared to those without, despite being on stable suppressive treatment [74,75]. Cognitive impairment and cognitive decline are also uncommon among individuals who initiated ART within the first year of infection. In a cross-sectional study of 26 PHI participants, only one was cognitively impaired after a median duration of 5.7 years of ART [76]. In the Sabes cohort, cognitive impairment was rare among participants with PHI over 192 weeks of follow-up [39], while the rate of cognitive decline was comparable between participants in the Chicago Early HIV Infection cohort participants and PWoH controls at the 2-year follow-up [40].

Ongoing neuronal injury and residual intracerebral immune activation post-ART also appear uncommon if ART is initiated during AHI. In the RV254 AHI cohort, the frequency of elevated CSF NfL at week 24 post-ART was 4% (1/24), compared to 44% (4/9) in CHI participants on suppressive ART [45]. Further, at 96 weeks post-ART, 22 RV254 participants demonstrated statistically comparable levels of CSF IL-6, neopterin, IP-10 and MCP-1 with 18 PWoH controls [41]. Early HIV treatment may also reduce the risk of CSF viral escape [17], which occurs in 5–10% of PWH on suppressive ART [18]. In the RV254 cohort, CSF escape was detected in 1% (1/90) and 0% (0/55) at weeks 24 and 96 post-ART [77]. While the long-term clinical relevance of CSF viral escape remains to be determined, CSF viral escape is associated with elevated levels of CSF immune activation markers even in asymptomatic individuals [78,79]. In this regard, the frequency of CSF viral escape in PHI settings is less clear. Nevertheless, one study reported that initiating ART during PHI resulted in lower levels of CSF HIV antibodies compared to those observed in CHI, supporting a lower level of intracerebral immune activation in the former [80].

Consistently, neuroimaging studies support a likely cessation of adverse CNS effects after early initiation of ART. For instance, MRS revealed a resolution of elevated Choline/Creatine ratio in the basal ganglia 6 months after initiating ART during AHI [54], and further brain volume loss was not observed among PWH who initiated ART within the first year of infection [50]. Another study examined the longitudinal MRS changes in 53 PHI participants, of whom 23 had initiated ART by the time of the follow-up scan [81]. It revealed a correlation between the duration of HIV infection and ratios of Choline/Creatine and Myo-inositol/Creatine in the frontal WM and parietal GM, suggesting progressive inflammation and gliosis. However, these changes were attenuated after the initiation of ART.

Taking pre- and post-ART neurological findings in early HIV studies into perspective, the first year of HIV infection appears to be a prime time of neuropathogenesis. For instance, evidence of HIV compartmentalization and neuronal injury has been observed in a subset of individuals during the first year of infection but not during AHI. While limited by the duration of follow-up, ART initiation during early HIV infection appears to halt the deleterious effects of HIV infection in the CNS. The benefits are most pronounced in RV254 participants who started ART during AHI. In addition to the absence of ongoing neuronal injury and persistent cognitive impairment, they demonstrate comparable levels of key immune activation markers in the CSF and inflammation-related metabolites in the MRS compared to PWoH during the initial years post-ART. However, these observations remain to be replicated in studies with larger sample sizes and longer periods of follow-up.

**Table 2:** Longitudinal CNS outcomes following ART initiation during acute and primary HIV infection.

**Cognitive Performance**	**AHI**	Trajectory analysis revealed improvement in cognitive test performance over 288 weeks of follow-up, with the greatest extent of improvement among those who performed worst during AHI [37].
	**PHI**	Compared to PWH who initiated ART during chronic infection, PWH who began ART during PHI demonstrated lower rates of cognitive impairment during post-ART assessment [39,76].
**Neuroimaging**	**AHI**	Evidence of neuronal damage, presented as a reduced N-acetyl aspartate/Creatine ratio in MRS, is absent during AHI and 24 weeks post-ART [54]. MRS further revealed a normalized Choline/Creatine ratio at 24 weeks after ART initiation.
	**PHI**	Ongoing brain volume loss was absent among PWH who initiated ART within the first year of infection [50]. Elevated Choline/Creatine and Myo-inositol/Creatine ratios in the frontal WM and parietal GM were attenuated after initiation of ART [81].
**CSF HIV Escape**	**AHI**	CSF HIV escape occurred in ~1% of PWH after ART initiation during AHI, compared to 5–10% in those who began ART during chronic infection [77].
	**PHI**	Data not available.
**CSF Biomarkers**	**AHI**	CSF NfL, a marker of neuronal injury, was not elevated during untreated AHI and 24 weeks post-ART [45]. Normalization of CSF IL-6, neopterin, IP-10, MCP-1 at 96 weeks post-ART [41].
	**PHI**	Data not available.

Abbreviations: AHI: Acute HIV Infection; ART: Antiretroviral Therapy; CSF: Cerebrospinal Fluid; MRS: Magnetic Resonance Spectroscopy; PHI: Primary HIV Infection.

## 8. Unaddressed Systemic Effects of HIV on Long-Term Neurological Outcomes after Early ART

While the CNS-related outcomes in RV254 are reassuring, it is important to note that systemic immunity is not completely restored following ART initiation during AHI. For instance, plasma immune activation and coagulation cascade activation markers, such as neopterin, IP10, MCP-1, D-dimer and hyaluronic acid, remained elevated in RV254 participants after 96 weeks of suppressive ART [41,82]. T-cell dysregulation, evidenced by an inverted blood CD4/CD8 ratio (i.e., <1), persists in 30–50% of RV254 participants [83,84]. A persistently inverted CD4/CD8 ratio is present in over two-thirds of PWH despite years of suppressive ART [85,86]. Further, it is associated with immunosenescence [87] and an increased risk of non-AIDS-related morbidities and mortalities [88], including cardiovascular complications [89].

In the general population, chronic inflammation is known to accelerate atherosclerosis [90]. Meanwhile, vascular risk factors and cardiovascular complications are known predictors of VCI that contribute to cognitive decline in aging populations, irrespective of underlying neurodegenerative diseases [8]. In this regard, a recent study examined whether ART initiation during AHI would reduce vascular risk when compared to CHI [91]. The study included 373 AHI (RV254) and 608 CHI cohort participants from Thailand who have been on an average of 6 years of ART. Based on the Framingham risk score [92], the two groups exhibited similar 10-year CVD risk after adjusting for age, sex and smoking status, raising questions about the potential benefits of early ART in reducing vascular risk factors. Taken together, while early ART possibly stalls the direct intracerebral pathogenesis of HIV, incomplete systemic immune recovery underscores the importance of conducting long-term outcome studies in early HIV cohorts to confirm the sustainability of neurological benefits, particularly from a vascular disease perspective.

## 9. Conclusions

Acute and early HIV infection studies have tremendously enhanced our understanding of HIV pathogenesis. They confirm that the CNS neuropathogenesis begins during AHI, mirroring what occurs in other compartments. Additionally, initiating ART during the first year of infection probably arrests the adverse effects of HIV in the CNS, resulting in better neuroprotection when compared to initiation during CHI. However, incomplete systemic immune recovery and immunologic dysfunction persist in PWH who initiated ART during AHI and likely in other early HIV infection settings. Future studies should confirm whether such residual systemic immune dysfunction is clinically relevant through long-term outcome evaluation in early HIV infection cohorts.

## Figures and Tables

**Table 1:** Viral and Serological Features of Fiebig I–V Acute HIV Infection.

I	HIV RNA positive; p24 antigen negative
II	p24 antigen negative; IgM negative
III	IgM positive; Western Blot negative
IV	Western Blot indeterminate
V	Western Blot positive without p31 protein band
